# Hierarchical bi-directional attention-based RNNs for supporting document classification on protein–protein interactions affected by genetic mutations

**DOI:** 10.1093/database/bay076

**Published:** 2018-08-21

**Authors:** Aris Fergadis, Christos Baziotis, Dimitris Pappas, Haris Papageorgiou, Alexandros Potamianos

**Affiliations:** 1 School of Electrical and Computer Engineering, National Technical University of Athens, Zografou Campus 9, Iroon Polytechniou str, Athens, Greece; 2 Institute for Language and Speech Processing, “Athena” Research and Innovation Center, Artemidos 6 & Epidavrou, Maroussi, Athens, Greece; 3 Department of Informatics, Athens University of Economics and Business, 76 Patission Str., Athens, Greece

## Abstract

In this paper, we describe a hierarchical bi-directional attention-based Re-current Neural Network (RNN) as a reusable sequence encoder architecture, which is used as sentence and document encoder for document classification. The sequence encoder is composed of two bi-directional RNN equipped with an attention mechanism that identifies and captures the most important elements, words or sentences, in a document followed by a dense layer for the classification task. Our approach utilizes the hierarchical nature of documents which are composed of sequences of sentences and sentences are composed of sequences of words. In our model, we use word embeddings to project the words to a low-dimensional vector space. We leverage word embeddings trained on PubMed for initializing the embedding layer of our network. We apply this model to biomedical literature specifically, on paper abstracts published in PubMed. We argue that the title of the paper itself usually contains important information more salient than a typical sentence in the abstract. For this reason, we propose a shortcut connection that integrates the title vector representation directly to the final feature representation of the document. We concatenate the sentence vector that represents the title and the vectors of the abstract to the document feature vector used as input to the task classifier. With this system we participated in the *Document Triage Task* of the BioCreative VI Precision Medicine Track and we achieved 0.6289 Precision, 0.7656 Recall and 0.6906 F1-score with the Precision and F1-score be the highest ranking first among the other systems.

Database URL: https://github.com/afergadis/BC6PM-HRNN

## Introduction

Precision medicine (PM) is an emerging area for diseases prevention and treatment that takes into account people’s individual variations in genes, environment and lifestyle (1). The PM Initiative intends to generate the scientific evidence needed to move the concept of PM into clinical practice (2). By extracting the ‘hidden’ knowledge in the scientific literature, we can help health professionals and researches in this PM challenge (3). Databases play a key role in this process by acting as a reference for the researchers and professionals (4). We are currently facing an exponentially increasing size of the biomedical literature which combined with the limited ability of manual curators to find the desired information, leads to delays in updated those databases with current findings. Currently the highest quality databases require manual curation, often in conjunction with support from automated systems (5).

Document classification attempts to automatically determine if a document or part of a document has particular characteristics of interest, usually based on whether the document discusses a given topic or contains a certain type of information. Accurate classification systems can be especially valuable to health professionals, researchers and database curators (6).

The BioCreative VI Track 4 ‘Mining protein interactions and mutations for PM’ provides a curated dataset that aims to leverage the knowledge available in the scientific published literature and extract useful information that links genes, mutations and diseases to specialized treatments (7). The PM tasks is a challenge consisting of two sub-tasks, namely the *Document Triage Task* ‘identify relevant PubMed citations describing genetic mutations affecting protein–protein interactions (PPI)’ and *Relation Extraction Task* ‘extract experimentally verified PPI affected by the presence of a genetic mutation’ task.

The automated document triage task is not new to the biomedical domain. In TREC 2004 Genomics Track one sub-task required the triage of articles likely to have experimental evidence warranting the assignment of Gene Ontology terms (8). The goal of this triage process was to limit the number of articles sent to human curators for more exhaustive and specific analysis. Also, in BioCreative II Task 2 (2007) the ‘Protein Interaction Article Sub-task 1’ is a document classification task for mining PPI from biomedical literature (9).

In this work, we present a deep learning system that participated in the *Document Triage Task* which calls for automatic methods capable of receiving a list of PMIDs (biomedical abstracts) and return a relevance-ranked judgement for triage purposes. The proposed system is a hierarchical bi-directional attention-based Re-current Neural Network (RNN) adapted to the biomedical domain. The results of our system on the above mentioned task are very promising and shows that deep learning systems can be succesfully applied to the biomedical domain.

## Related work

Machine learning algorithms have been widely and successfully used in order to extract knowledge from big data in bioinformatics. Some well-known algorithms, e.g. Naive Bayes, Support Vector Machines and Random Forests among others, have been applied in biomedical literature triage (10), genomics (11), genotypes-phenotypes relations (12) and numerous other domains (13). Sparse lexical features such as bag-of-words, n-grams, word frequencies (term-frequency and/or inverse-document-frequency) and hand-crafted features are used to train those algorithms (14).

Recently, deep-learning systems have become popular in learning text representations, mostly two variants of them, Convolutional Neural Networks (CNN) and RNNs. Although CNNs have been successfully used in text classification (15–18), RNNs have produced excellent results processing text (19–24), especially the variants Long Short-Term Memory (LSTM) (25) and Gated Re-current Units (GRU) (26). RNNs are designed to utilize sequential information. This sequential nature is suitable to process varying length input data such as speech and text. However, there are many cases where both past and future inputs affect output for the current input. For these cases, Bi-directional Re-current Neural Networks (BRNNs) have been designed and used widely (27).

Tang *et al*. (19) introduce a neural network that learns vector-based document representations. In this hierarchical model, the first level learns sentence representation using a CNN or a LSTM network and the second level uses GRUs to encode this sentence information into a document representation. Yang *et al*. (20) use a hierarchical attention LSTM network for document classification. The attention layers applied at the word and sentence-level, capture the most important content leading to better document representation. Zhou *et al*. (22) have exploited bi-directional LSTM with attention layer for relation classification. Also Zhou *et al*. (23), instead of using the attention mechanism to produce the sentence and document vectors, they apply a two-dimensional pooling operation over the two dimensions of the network (time-step and feature vector) in order to produce more meaningful features for sequence modelling tasks. Liu *et al*. (21), based on the same hierarchical principle, use the multi-task learning framework to improve the performance of their model in text classification and other related tasks. Also, Zhang *et al*. (24) propose a multi-task learning architecture with four types of re-current neural layers for text classification. Baziotis *et al*. (28), successfully applied a two-level bi-directional LSTM with an attention mechanism for message-level sentiment analysis on Twitter messages at SemEval-2017 Track 4 (29).

Our work is mostly influenced by (20, 22) and is very similar to (28). We employ a hierarchical bi-directional GRU (HBGRU) network equipped with attention layers which generates dense vector representations for each document and uses those representations as features for classification. We adapt our model on the specific features of the domain by proposing a shortcut connection that integrates the title vector representation directly to the final feature representation of the document. This shortcut connection improves the performance of the model on the BioCreative VI PM dataset.

## System description

**Figure 1.**
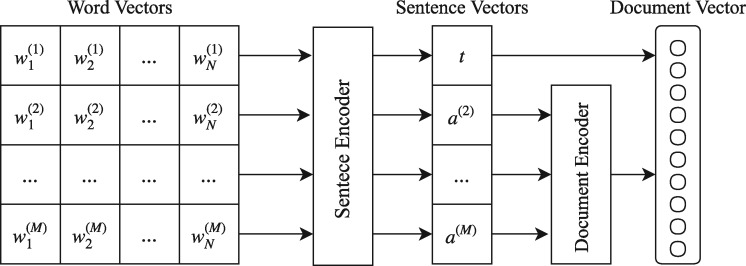
Overview of our proposed system. *Word Vectors* is a matrix of word embeddings, where *M* is the maximum number of sentences and *N* the maximum number of words in a sentence. *t* refers to the *Sentence Encoder* representation for the *title* vector and *a*^(2)^, … , *a*^(M)^ to the representations of the *abstract* vectors.

The model we propose is a hierarchical bi-directional RNN network as shown in Figure 1. We equip the RNN layers with an attention mechanism for identifying the most informative words and sentences in each document. The first level consists of an RNN that operates as a sentence encoder reading the sequence of words in each sentence and producing a fixed vector representation (sentence vector). Then, a second level RNN operates as a document encoder reading the sequence of sentence vectors of the abstract and producing a vector representation (document vector). We argue that the title of the citation itself usually contains important information more salient than a typical sentence in the abstract. For this reason, we propose a shortcut connection that integrates the title vector representation directly into the document vector representation. This concatenated vector is used as a feature vector for classification. We add a fully-connected layer with a sigmoid activation function for performing binary classification.

### Text pre-processing

As a first pre-processing step we perform sentence segmentation and tokenization splitting the document in constituent sentences and tokens. We use Punkt sentence and word tokenizers of the Natural Language Toolkit as a sentence splitter and word tokenizer, respectively (30).

### Annotations

In order to incorporate domain knowledge in our system, we annotate all biomedical named entities namely *genes, species, chemical, mutations* and *diseases*. Each entity mention is surround by its corresponding tags as in the following example:

Mutations in <species>human</species> <gene>EYA1</gene> cause <disease>branchio-oto-renal (BOR) syndrome</disease> …

The annotations are obtained using the provided RESTful API of PubTator, a Web-based text mining tool for assisting Biocuration (31–33).

### Input layer

We represent each document as a matrix $A\in R^{M\times N}$, where *M* is the maximum number of sentences that a document may have and *N* is the maximum number of words a sentence may have. We embed the words *w* to a low-dimensional vector space through an embedding layer of size $E,\;w\in R^{E}$. A sentence *S* consists of a sequence of *N* words $S=(w_{1},w_{2},\ldots ,w_{N}),\;S\in R^{N\times E}$. The embedding layer weights are initialized with the pre-trained word embeddings provided by (34). These word embeddings are trained on PubMed articles and PMC full text papers using word2vec (35) with the skip-gram model and a window size of 5. The dimensionality of the word vectors is 200. Out of vocabulary words, for which we do not have a word embedding, are mapped to a common **<unk>** (unknown) token. Unknown token along with named entities starting and closing tags, get distinct word embeddings by sampling from a uniform distribution with range (−0.05,0.05).

### Sentence encoder

**Figure 2.**
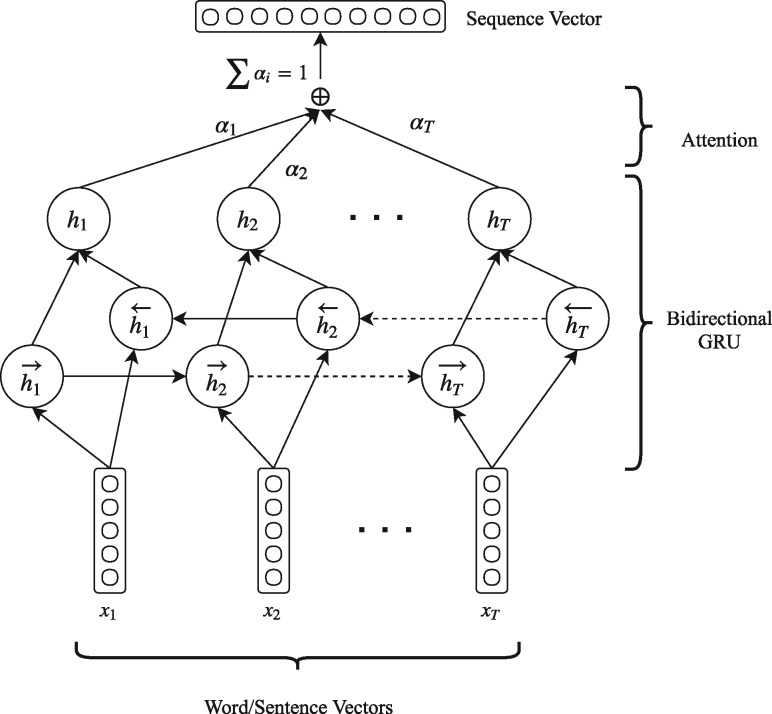
Architecture of our proposed sequence encoder. The same architecture is used for encoding a sequence of word vectors to a sentence vector (sentence encoder) and a sequence of sentence vectors to a document vector (document encoder). When used as a sentence encoder *x* represent words, *T* takes values up to *N* and the output sequence vector is a sentence vector. When used as a document encoder *x* represent sentences, *T* takes values up to *M* and the output is a document vector.

After embedding the words to the low-dimensional semantic space we use the sequence encoder in order to obtain a vector representation for each sentence. The sequence encoder consists of a bi-directional GRU with an attention layer that reads the sequence of word vectors of each sentence and produces a sentence vector. The architecture of the sequence encoder is shown in Figure 2.

A GRU takes as input the sequence of word vectors of a sentence and produces a sequence of word annotations (output), $H=(h_{1},h_{2},\ldots ,h_{N})$, where $h_{j},j\in [1..N]$ is the hidden state of the GRU at time-step *j*, summarizing all the information of the sentence up to *w_j_* word. We use bi-directional GRU in order to capture the contextual information of the words from both their left and their right context. A BGRU consists of a forward GRU that reads the sentence from *w*_1_ to *w_N_* and a backward GRU that reads the sentence from *w_N_* to *w*_1_. We obtain the final annotation for each word *w_j_* by concatenating the annotations from both directions.

$$h_{j}^{\left(i\right)}=\vec{h_{j}^{\left(i\right)}}∥\overset{\leftarrow }{h_{j}^{\left(i\right)}},\;j\in [1\ldots N],\;h_{j}^{\left(i\right)}\in R^{2S}$$

where ∥ denotes concatenation, $\vec{h_{j}^{\left(i\right)}}$ and $\overset{\leftarrow }{h_{j}^{\left(i\right)}}$ are the hidden states for the forward and backward GRU, respectively, of *i*-th sentence at time-step *j* and *S* the size of the sentence-level GRU layer.

We use an attention layer in order to identify the most informative words in each sentence and enforce their contribution to the final sentence vector. The attention layer assigns a weight $\alpha_{j}^{\left(i\right)}$ to each word annotation $h_{j}^{\left(i\right)}$. The sentence vector $v^{\left(i\right)}$, which is the vector representation of the *i*-th sentence, is computed as the weighted sum of all the word annotations $h_{j}^{\left(i\right)}$.

$$\begin{array}{l} v^{\left(i\right)}=\sum_{j=1}^{N}\alpha_{j}^{\left(i\right)}h_{j}^{\left(i\right)},\;i\in [1\ldots M],\;v^{\left(i\right)}\in R^{2S}\\ \alpha_{j}^{\left(i\right)}=\frac{\;\text{exp}(e_{j}^{\left(i\right)})}{\sum_{t=1}^{N}\;\text{exp}\;(e_{t}^{\left(i\right)})},\;j\in [1\ldots N]\\ e_{j}^{\left(i\right)}=\tanh(W_{w}h_{j}^{\left(i\right)}+b_{w}) \end{array}$$

where *W_w_*, *b_w_* are the attention layer weights and bias and $v^{\left(i\right)}$ is the vector representation of the *i*-th sentence.

Moreover, we denote the sentence vector of the title as $t=v^{\left(1\right)}$ and the sentence vectors of the abstract as $a^{\left(i\right)}=v^{\left(i\right)},i\in [2\ldots M]$ as in Figure 1.

### Document encoder

Having the vector representations for each sentence, we feed them to the document encoder in order to obtain the final vector representation for the whole document. Notably, we do not feed the vector of the title *t* to the sentence encoder, but only the vectors of the abstract *a_i_*. Instead of feeding the title vector *t* in the document encoder with the rest of the sentence vectors (abstract), we create a shortcut connection by integrating it directly to the final document feature vector *d*. We hypothesize that the title of a paper contains concentrated information which will be diluted if passed in the document encoder with the other sentences, even with the addition of the attention mechanism. By integrating title vector *t* directly into the document feature vector *d* we keep the title information intact. The remaining sentence vectors are fed into the document encoder in order to get the vector representation of the whole abstract *a*. The architecture of the document encoder which is identical to the sentence encoder is shown in Figure 2.

Similar to the sentence encoder, we use a BGRU in order to get annotations for each abstract vector *a_j_* summarizing the information form the sentences around sentence *j*.

$$h_{j}=\vec{h_{j}}∥\overset{\leftarrow }{h_{j}},\;j\in [1\ldots M],\;h_{j}\in R^{2D}$$

where ∥ denotes concatenation, $\vec{h_{j}}$ and $\overset{\leftarrow }{h_{j}}$ are the hidden states for the forward and backward GRU, respectively, at time-step *j*, *M* the number of abstract vectors and *D* the size of the document-level GRU layer. We use an attention layer in order to identify the most informative sentences of the abstract and enforce their contribution to the final vector representation *a*. The attention layer assigns a weight *α_j_*, to each sentence annotation and we aggregate them by computing the weighted sum of all the sentences annotations.

$$\begin{array}{c} a=\sum_{j=1}^{M}\alpha_{j}h_{j},\;a\in R^{2D}\\ \alpha_{j}=\frac{\;\text{exp}(e_{j})}{\sum_{t=1}^{M}\;\text{exp}\;(e_{t})}\\ e_{j}=\tanh(W_{a}h_{j}+b_{a}) \end{array}$$

where *W_a_*, *b_a_* are the layer weights and bias.

### Output layer

The final document vector *d* is computed by concatenating the representations of title and abstract vectors

$$d=t∥a,\;d\in R^{2S+2D}$$

The output layer is a fully connected layer with single neuron and a logistic (sigmoid) activation function that performs the binary classification (logistic regression). It uses the document vector representation *d* as feature vector to predict the probability of the two classes.

## Experiments and results

### Dataset

We evaluate our system on the dataset provided by the BioCreative VI (BC6) Precision Medicine Track (PM), Document Triage Task (7). This training dataset consists of 4082 training biomedical abstracts which are classified as ‘relevant’/’no relevant’ when the article mentions or not PPIs influenced by genetic mutations. The test dataset consists of 1427 abstracts. The number of relevant abstracts is 1729 (42.36%) in the train set and 704 (49.33%) in the test set (Table 1).

**Table 1.** Dataset of BC6-PM document triage task

Dataset	Negative	Positive	Total
Train	2353 (57.64%)	1729 (42.36%)	4082
Test	723 (50.67%)	704 (43.33%)	1427

### Text pre-processing

**Figure 3.**
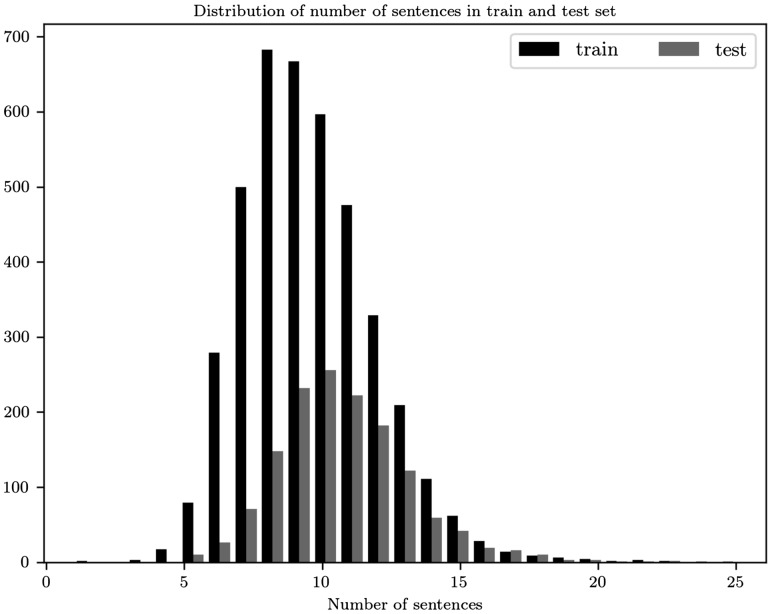
Distribution for the number of sentences in the abstracts of the train and test sets.

Our model, as described, is a sequence encoder which on the first level reads a documents that we represent as matrices $A\in R^{M\times N}$. To choose the values of *M* and *N* we explore the distribution of the sentences in the abstracts of the train set. The maximum number of sentences is 23, which we set as the value of *M*. In the test set 99.86% have less or equal to 23 number of sentences. For comparison reasons in Figure 3, we display the distribution for both train and test sets. Also, as a pre-processing step we remove stop words and punctuation when these tokens are not part of a biomedical entity.

**Figure 4.**
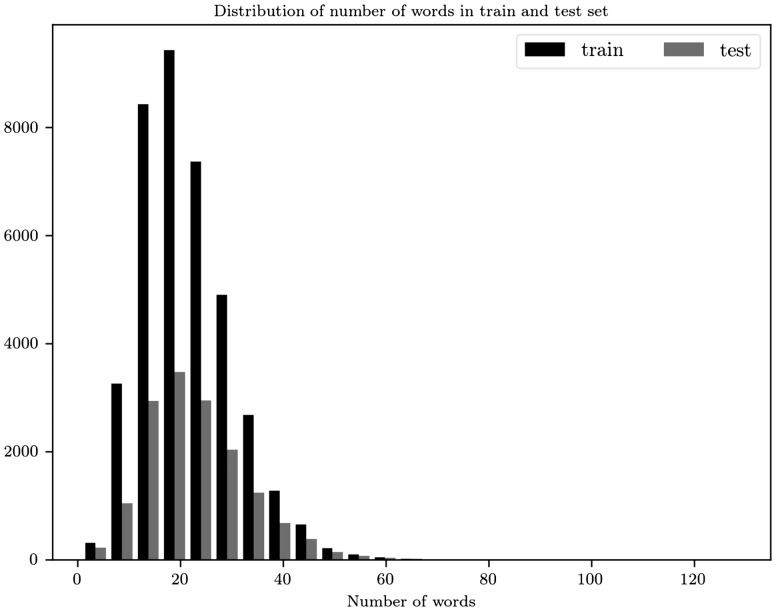
Distribution for the number of words per sentence for the train and test sets.

Examining the distribution of the number of words in sentences (Figure 4) we choose 45 to be the maximum words per sentence. 98.63% sentences of train and 97.51% of test abstracts have less or equal to 45 words per sentence. At the end each document is represented as a matrix $A\in R^{23\times 45}$. We use zero padding, appended to the end of a sequence, both in documents and sentences in order to have the same number of sentences and words, respectively.

### Model training

Neural networks are notoriously prone to over-fitting (36). For this reason, we adopt a series of measures in order to regularize our model. First, we add Gaussian noise to the input (embedding) layer to limit the amount of information that can be stored in a network (37). This means that practically the network never sees the exact same sentence more than once during training. Distortion of the training data can be considered as a data augmentation technique. We add noise by sampling from a zero-mean Gaussian distribution at each batch.

We use dropout to the layers of the network as another over-fitting restricting technique. Dropout randomly disables a certain proportion of the neurons in a layer on each training example (or batch). For each training example a sub-part of the network is trained. Dropout improves the network performance because it forces each neuron to learn disentangled features. This way the network learns to recognize the same patterns in multiple ways, which leads to a better model (38). We apply dropout on the embedding layer on the sentence and document encoders both on their BGRU layers and their attention layers.

Many methods have been used to improve stochastic gradient descent such as momentum, annealed learning rates and *L*_2_ weight decay. As an optimizer, we use Adam (39) with the standard deterministic cross-entropy objective function. We add *L*_2_ penalty to the objective function to prevent large weights and we clip the norm of the gradients at 5 to avoid exploding gradients (40).

As a last step, we perform early-stopping. We stop the training of the network when the F1-score of the development set stops increasing for a certain number of epochs (41). We monitor the change of F1-score instead of the loss of the development set because its the official evaluation metric used and this way we directly optimize our model for the task. If F1-score does not improve (increase) from the last best value for 6 epochs, the training is stopped and the last best model is kept.

Hyper-parameter tuning in neural networks is a very challenging process. In addition to the time consuming training of the neural network, usually we have to tune a lot of hyper-parameters, which are highly correlated (e.g. increasing the number of neurons changes the optimal dropout rate). As it has been shown in (42), grid search is very inefficient and random search finds consistently better models. However, in our work we adopt the Bayesian optimization approach (43) in order to perform a smart search in the high-dimensional hyper-parameter space. This way we obtain a set of reasonable hyper-parameters in a very small number of trials. Table 2 shows the optimal hyper-parameter values that we obtained. To choose the hyper-parameters we split the training set to *training, development* and *evaluation*, using 80%, 10% and 10% of the dataset, respectively. For the training of the final model, to get the predictions for the test set, we split the training set to *training* and *development*, using this time 95% and 5% of the dataset.

**Table 2.** Hyper-parameter values of our model

	Layer	Size	Dropout	Noise (σ)
	Embedding	200	0.2	0.2
**Sentence encoder**	GRU	150 (x2)	0.3	—
	Attention	1	0.3	—
**Document encoder**	GRU	150 (x2)	0.3	—
	Attention	1	0.3	—

### Experimental setups

Our first experimental setup was to test the impact of the shortcut connection. Testing with our training, development and evaluation sets the model with the shortcut connection gave better performance. Our hypothesis that the model benefits from the shortcut connection is also supported by the official results described in the following section.

Also, after the competition of the completion, we wanted to investigate the impact of incorporating domain knowledge to the model by annotating biomedical entities. The fact that we can use word vectors either for entity tokens or for entities as multi-word expressions (MWEs) lead as to investigate the impact of different tokenization options. So, the parameters we tune for our new experiments are the inclusion or not of the annotations of the biomedical entities and the tokenization options as explained below. The capitalization of the words is retained and we remove stop words and punctuations. Compared with the model that participated in the competition the pre-processing was different in that we kept the stop words and converted words to lower case.

The tokenization described hereafter is applied to mentions of biomedical entities only. We investigate three options. The first (Tokens) is to tokenize the entity as all other tokens. This results in removing punctuation, if any, used between entity tokens. As a second option we choose to keep these mentions as MWEs and tokenize then by spaces. In this way, we keep the punctuation between words. The third option (Both) is to tokenize the entity and also insert the multi-word version of it. In Table 3, we give a tokenization example for the disease *brancio-oto renal (BOR) syndrome* with the three options.

**Table 3.** Tokenization options for biomedical entity mentions [e.g. ‘brancio-oto-renal (BOR) syndrome’]

Option	Result
Tokens	‘branchio’, ‘oto’, ‘renal’, ‘BOR’, ‘syndrome’
MWE	‘branchio-oto-renal’, ‘(BOR)’, ‘syndrome’
Both	‘branchio-oto-renal’, ‘(BOR)’, ‘syndrome’, ‘branchio’, ‘oto’, ‘renal’, ‘BOR’, ‘syndrome’

When we use the MWE of an entity we get one word embedding for entities like *autosomal-dominant* and two word embeddings when tokenized: *autosomal*, *dominant*. We hypothesize that the MWE will have better semantics captured by its word embedding. The third option covers cases where the MWE has no word embeddings. For example, the chemical *p-Benzoyl-L-phenylalanine* as a MWE does not have an embedding in our word vectors, but all its tokens: *‘p’, ‘Benzoyl’, ‘L’, ‘phenylalanine’*, have. As a last step, when we use the option to keep the tokens and the MWE, when the two match we keep only the tokens version. For instance, the disease *Rieger Syndrome* as a MWE and as tokens give the same result: *‘Rieger’, ‘Syndrome’*.

### Results

We submitted three runs for the competition. The official results are shown in Table 4. In this table, we also display the baseline given by the organizers, as well as our own baseline computed using a SVM model. For the first two runs, we do not use the proposed shortcut connection but we chance the RNN size keeping the other hyper-parameters unchanged. The increase of the RNN size gave an increase to the F1-score. For the third run, we keep the larger RNN size and apply the shortcut connection. The results shows that the model benefits from the proposed approach.

**Table 4.** Official results for the submitted runs along with the organizer’s baseline and an SVM model

Model	Run	RNN size	Shortcut connection	Precision	Recall	F1-score
Baseline	—	—	—	0.6122	0.6435	0.6274
SVM	—	—	—	0.5850	0.7869	0.6711
HBGRU	1	100	No	0.6136	0.7670	0.6818
	2	150	No	0.5944	0.8139	0.6871
	3	150	Yes	0.6289	0.7656	**0.6906**

The hyper-parameters not mentioned remain unchanged.

To study the affect of annotation and tokenization, we perform a 5-fold cross validation on the train dataset. We display the F1-scores in Table 5. For the two options, to annotate or not the biomedical entities we use the three aforementioned tokenization options. We test the null hypothesis that there is no statistical significant difference between the scores we performed a two-way mixed factorial ANOVA test. In the present case, the Mauchly’s test indicates that there is no evidence of heterogeneity of covariance, $x^{2}=2.463;\;p=0.292$. The ANOVA test showed that there is no statistical significant difference within-subjects factors (tokenization options), F(2,16)=1.953; p=0.174, nor between-subjects factors (annotation), F(1,8)=0.10; p=0.925. Based on these results we accept the null hypothesis.

**Table 5.** F1-scores of the 5-fold cross validation with options to annotate or not biomedical entities and the three tokenization options

		Tokenization options		
Fold	Annotation	Tokens	MWE	Both
1	Yes	0.6078	0.6097	0.6088
2	Yes	0.7493	0.7550	0.7399
3	Yes	0.8023	0.7883	0.8067
4	Yes	0.7834	0.7846	0.7581
5	Yes	0.6974	0.7019	0.7018
*Average*		**0.7280**	**0.7279**	**0.7231**
1	No	0.6257	0.6171	0.6178
2	No	0.7557	0.7516	0.7555
3	No	0.7988	0.7903	0.8012
4	No	0.7904	0.7682	0.7578
5	No	0.7145	0.7136	0.7037
*Average*		**0.7370**	**0.7282**	**0.7272**

## Conclusions and future work

One of the tasks that help PM Initiative to its goal is the mining of biomedical literature mentioning PPIs changed by genetic mutations. In this paper, we describe our proposed system that participated in such a challenge organized by BioCreative and launched as ‘BioCreative VI Track 4: Mining protein interactions and mutations for PM. We participated in the *Document Triage Task* of the competition building hierarchical bi-directional attention-based RNNs. In our system, we modify the typical RNN model by adding a shortcut connection between the title vector and the final feature representation of the document. The hypothesis we test is that the title of the paper itself usually contains important information more salient than a typical sentence in the abstract. The shortcut connection increased the performance of the model as shown in Table 4 achieving 0.6289 Precision, 0.7656 Recall and 0.6906 F1-score with the Precision and F1-score be the highest in the challenge’.

To further investigate options that might improve the performance of our model, we choose to incorporate domain knowledge by annotating biomedical entities. Annotations are very useful to tasks such as Named Entity Recognition and Relation Extraction (22). The motivation to add annotations to a document classification task was that the attention layer would benefit from them. The treatment of the named entities as MWE or tokens or inserting both in a sentence lead us to different tokenization options. Our results suggest that the RNN model is capable to capture contextual information from the text without the need of the annotations and independently of the tokenization options in the particular dataset.

The result of no statistical significant difference may be due to two factors. One factor is the way we choose to annotate entities using positional indicators (tags) which might not be suitable for this task. The other factor is related to the word embeddings we use to initialize the embeddings layer. We hypothesize that the training data for the word embeddings do not have enough mentions for the MWEs of the named entities in order to capture the appropriate syntactic and semantic informations and that the embeddings of individual tokens of named entities might not carry the desirable semantics.

In future work, we plan to train our word embeddings on PubMed articles and to investigate other options to annotate named entities. Training our word embeddings will allow us to align the pre-processing step on both the training corpus and dataset reducing out of vocabulary words. About the annotation options one alternative is to use the BIO tags. We can create vectors that will represent the annotations *O, B-disease, I-disease, B-gene, I-gene* and so forth. These vectors can be concatenated to the word embeddings of all tokens according to their annotations.

## Acknowledgements

We acknowledge support of this work by the project “Computational Science and Technologies: Data, Content and Interaction” (MIS 5002437) which is implemented under the Action “Reinforcement of the Research and Innovation Infrastructure”.

## Funding

Funding by the Operational Programme “Competitiveness, Entrepreneurship and Innovation” (NSRF 2014-2020) and co-financed by Greece and the European Union (European Regional Development Fund).

*Conflict of interest*. None declared.
